# MicroRNA-6862 inhibition elevates sphingosine kinase 1 and protects neuronal cells from MPP^+^-induced apoptosis

**DOI:** 10.18632/aging.202335

**Published:** 2020-12-19

**Authors:** Gang Xue, Ju-ping Chen, Ya Li, Zhi-qing Zhang, Jian-liang Zhu, Wanli Dong

**Affiliations:** 1 Department of Neurology, The First Affiliated Hospital of Soochow University, Suzhou, China; 2 Department of Neurology, Fengcheng Hospital of Fengxian Distric, Shanghai, China; 3 Department of Neurology, Changshu Hospital Affiliated to Nanjing University of Chinese Medicine, Changshu, China; 4 The Central Laboratory, North District, Suzhou Municipal Hospital Affiliated to Nanjing Medical University, Suzhou, China; 5 Institute of Neuroscience, Soochow University, Suzhou, China; 6 Department of Emergency and Intensive Care Unit, The Second Affiliated Hospital of Soochow University, Suzhou, China

**Keywords:** neuronal cells, MPP+, microRNA-6862 and SphK1

## Abstract

MPP^+^ (1-methyl-4-phenylpyridinium)-induced dopaminergic neuronal cell apoptosis is associated with sphingosine kinase 1 (SphK1) inhibition. We here tested the potential effect of microRNA-6862 (miR-6862), a novel SphK1-targeting miRNA, on MPP^+^-induced cytotoxicity in neuronal cells. MiR-6862 locates in the cytoplasm of SH-SY5Y neuronal cells. It directly binds to *SphK1* mRNA. In SH-SY5Y cells and HCN-2 cells, ectopic overexpression of miR-6862 decreased SphK13’-untranslated region luciferase reporter activity and downregulated its expression. miR-6862 inhibition exerted opposite activity and elevated SphK1 expression. In neuronal cells, MPP^+^-induced cell death was significantly inhibited through miR-6862 inhibition. Conversely, ectopic overexpression of miR-6862 or CRISPR/Cas9-induced SphK1 knockout augmented MPP^+^-induced apoptosis in the neuronal cells. Importantly, antagomiR-6862 failed to inhibit MPP^+^-induced apoptosis in SphK1-knockout SH-SY5Y cells. These results suggest that inhibition of miR-6862 induces SphK1 elevation and protects neuronal cells from MPP^+^-induced cell death.

## INTRODUCTION

Currently, Parkinson's disease (PD) is the second most common neurodegenerative disease[1, 2]. It affects over seven million people globally [2]. PD is characterized by a progressive loss of dopaminergic (DA) neurons in the midbrain [3–6]. Sustained and unsolved oxidative stress could be one primary cause of DA neuronal cell death. It is also known as the primary pathogenesis mechanism of PD [7].

Disruption of mitochondrial respiratory chain in DA neurons would lead to robust radical oxidative species (ROS) production and oxidative injury. This will then cause calcium overload, lipid peroxidation, DNA breaks, and protein aggregation [7–9]. These events together lead to DA neuronal cell death [7]. The mitochondrial respiratory chain complex inhibitor 1-methyl-4-phenyl-1,2,3,4-tetrahydropyridine (MPTP) has been applied to establish animal PD models [7]. In the brain of primates, MPTP is converted into MPP^+^ (1-methyl-4-phenylpyridinium) by monoamine oxidase B (MAO-B), causing parkinsonism by killing DA neurons in substantia nigra [7]. Additionally, as a commonly-utilized cellular model of PD, MPP^+^ is added to the cultured neurons and neuronal cells [10–13].

Sphingosine kinase 1 (SphK1) phosphorylates sphingosine to sphingosine-1-phosphate (S1P) to promote cell survival [14–16]. Conversely, SphK1 inhibition, silencing, or knockout would induce sphingosine/ceramide accumulation and S1P depletion that lead to cell death [16]. SphK1 is expressed in neurons and progenitor cells, and is enriched in cellular membranes of endocytosis, synapses, and mitochondria [17]. Neuronal SphK1 expression is decreased in Alzheimer’s disease (AD) brain, indicating its potential role in the pathogenesis of AD. Upregulation of neuronal SphK1 could promote microglia-induced phagocytosis of aggregated amyloid β (Aβ) and improve cognitive deficits [18]. SphK1-S1P signaling is essential in regulating sensory ganglia development and survival of neurons and progenitor cells [19]. In motor neurons, SphK1 functions in Nrf2 signaling cascade to regulate neuropeptide biogenesis and secretion [19].

Studies have shown that MPP^+^ serve as a main contributor of subsequent neuronal cell apoptosis as it downregulated SphK1 expression and inhibited its activity [20, 21]. Conversely, SphK1 activation by exogenously adding S1P protected neuronal cells from MPP^+^-induced cell apoptosis [22]. Therefore, restoring SphK1expression/activity could protect neuronal cells from MPP^+^ [20, 21].

microRNAs (miRNAs) is a large family of small non-coding RNA molecules containing ~22 nucleotides [23, 24]. miRNAs are capable of silencing targeted genes through a post-transcriptional mechanism [23, 24]. Specifically, miRNAs function via base-pairing with the complementary sequences in the 3’-untranslated region (UTR) of targeted mRNAs/genes. This will cause the degradation and/or translation inhibition of targeted genes [23, 24]. Several SphK1-targeting miRNAs have been identified, and many of them are in cancer cells [25–27]. Here we identifiedmicroRNA-6862 (miR-6862) as a novel SphK1-targeting miRNA. The current study is to determine whether miR-6862 would affect SphK1 expression in neuronal cells, and if so, whether miR-6862 inhibition would protect neuronal cells from MPP+-induced damage.

## RESULTS

### miR-6862 directly binds to and silences SphK1 in SH-SY5Y neuronal cells

First, the microRNA database TargetScan (V7.2) [28] was consulted to identify miRNAs that can putatively bind to *SphK1* 3’-UTR [28]. A total of 144 different miRNAs complementary pairing with *SphK1* 3’-UTR were retrieved,. Eight (8) of them have a context^++^ score less than -0.5 (Table 1) and context++ score percentile of over 95% (Table 1). These parameters indicated a high percentage of direct binding between these proposed miRNAs and *SphK1* 3’-UTR [28]. Next, each of the eight miRNA mimics (500 nM for 48h) was individually transfected to SH-SY5Y neuronal cells. Among which microRNA-6862-3p (miR-6862) resulted in the most significant *SphK1* mRNA reduction. Thereafter, miRbase (v21.0) and miRDB databases showed that there were over 250 predicted gene targets of miR-6862. Among which, SphK1 is the top 10% most possible targets.

**Table 1:** miRNAs complementary pairing with SphK1 3’-UTR with context++ score less than -0.5.

**No.**	**miRNA**	**Position**	**Seed match**	**Context^++^ score**	**context^++^ score percentage**
1	hsa-miR-3677-3p	235-242	8mer	-0.78	99
2	hsa-miR-6862-3p	113-120	8mer	-0.64	99
3	hsa-miR-6716-5p	262-269	8mer	-0.61	99
4	hsa-miR-6784-3p	113-120	8mer	-0.6	99
5	hsa-miR-4651	231-238	8mer	-0.59	98
6	hsa-miR-608	231-238	8mer	-0.56	98
7	hsa-miR-5004-3p	246-253	8mer	-0.52	99
8	hsa-miR-4505	266-272	7mer-m8	-0.5	99

**Figure 1:**
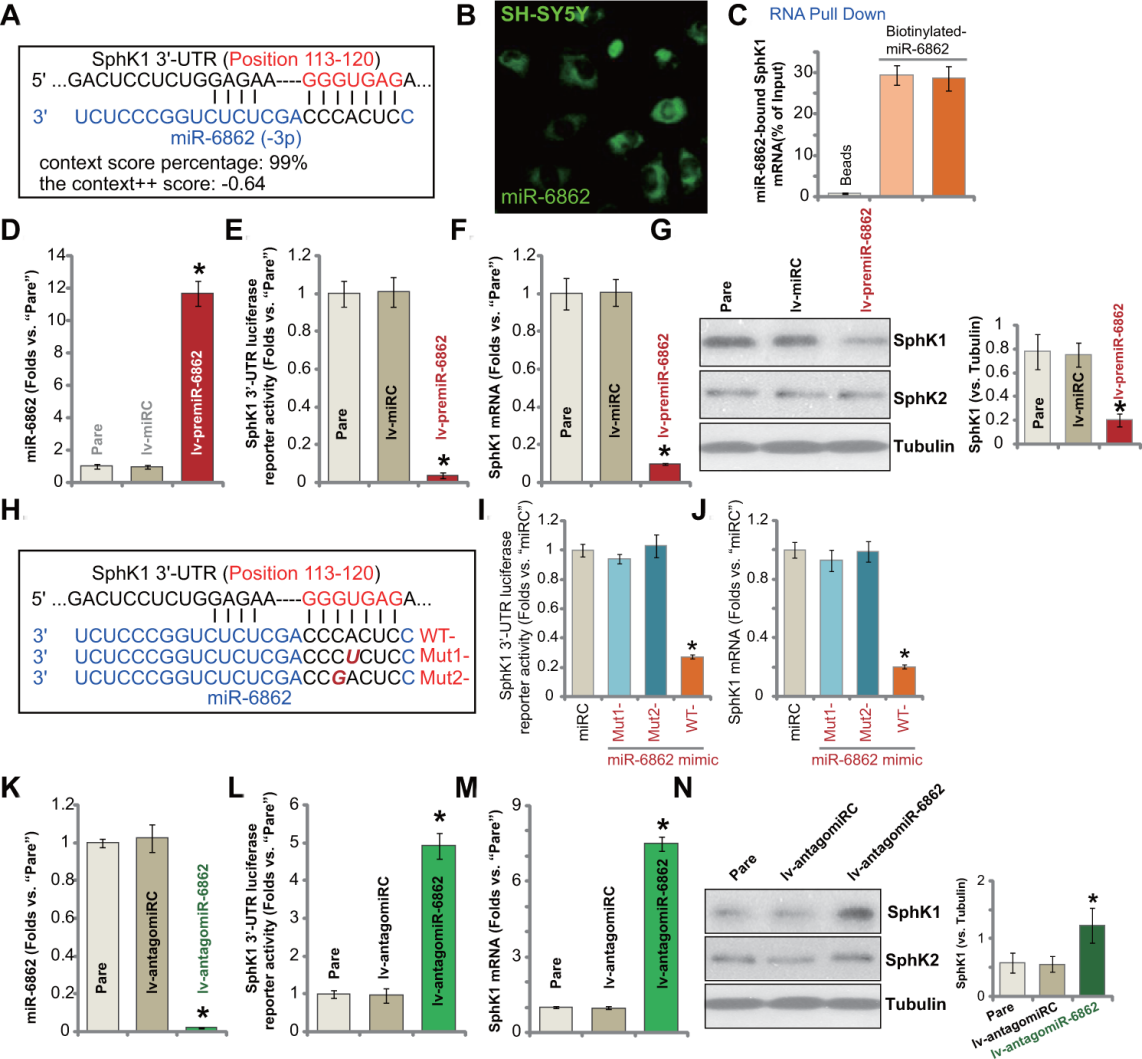
**miR-6862 directly binds to and silences SphK1 in SH-SY5Y neuronal cells.** miRNA-6862 putatively targets *SphK1 mRNA* 3’-UTR (untranslated region, at position of 113-120) (**A**). miRNA-6862 (fluorescence-tagged) locates in the cytoplasm of SH-SY5Y cells (**B**). RNA pull down showed a directing binding between biotinylated-miR-6862 and *SphK1 mRNA* in SH-SY5Y cells (**C**); Stable SH-SY5Y cells with the lentiviral construct encoding the premiR-6862 sequence (lv-premiR-6862) or the nonsense miRNA sequence (lv-miRC) were established, expression of listed genes (mRNA and protein) was shown (**D**, **F**, **G**). The relative SphK1 3’-UTR luciferase reporter activity was tested as well (**E**). SH-SY5Y cells were transfected with 500 nM of wile-type (“WT-“) or the mutant (“Mut1-”/“Mut2-”) miRNA-6862 mimics (sequences were listed in **H**), control cells were transfected with nonsense control miRNA (“miRC”), after 48h the relative SphK1 3’-UTR luciferase reporter activity (**I**) and *SphK1* mRNA expression (**J**) were tested. Stable SH-SY5Y cells with the lentiviral construct encoding the anti-sense of premiR-6862 (lv-antagomiR-6862) or the anti-sense control sequence (lv-antagomiRC) were established, expression of listed genes was shown (**K**–**N**). The relative SphK1 3’-UTR luciferase reporter activity was tested as well (**L**). “Pare” stands for the parental control cells (same for all Figures). Data were presented as mean ± standard deviation (SD, n=5). * *P* < 0.05 vs. “lv-miRC”/“miRC”/“lv-antagomiRC” cells. Experiments in this figure were repeated five times with similar results obtained.

miR-6862 putatively targets the *SphK1* 3’-UTR (at position 113-120, Figure 1A). The context score percentage of miR-6862-*SphK1* 3’-UTR binding is 99%. The context^++^ score is-0.64 (TargetScan V7.2 [28], Figure 1A). Figure 1B demonstrated that miR-6862 fluorescence mainly located in the cytoplasm of SH-SY5Y neuronal cells. Some was in cell nuclei (Figure 1B). Furthermore, RNA-Pull down assay [28] results, Figure 1C, demonstrated that the biotinylated-miR-6862 directly bound to *SphK1* mRNA in SH-SY5Y cells (Figure 1C). These results suggested a direct binding between miR-6862 and *SphK1* mRNA in neuronal cells.

To test whether miR-6862 can affect SphK1 expression, a lentiviral construct encoding the premiR-6862 sequence (see Table 2) was established. The construct, lv-premiR-6862, was transduced to SH-SY5Y neuronal cells. Via puromycin-mediated selection stable cells were established. The qPCR assay results in Figure 1D demonstrated that the mature miR-6862 expression increased over ten folds (*vs.* parental control cells) in lv-premiR-6862-expressing SH-SY5Y cells. Subsequently, SphK1 3’-UTR luciferase reporter activity decreased over 90% (Figure 1E). *SphK1* mRNA expression was dramatically downregulated as well (Figure 1F). Testing SphK1 protein expression by Western blotting confirmed SphK1 protein downregulation in lv-premiR-6862-expressing SH-SY5Y cells (Figure 1G). SphK2 protein expression, however, was unchanged (Figure 1G). The lentiviral construct encoding nonsense miRNA sequence, lv-miRC, did not change the expression of miR-6862 and SphK1 in SH-SY5Y neuronal cells (Figure 1D–1G).

**Table 2:** Sequences in this study.

**Genes**	**Forward sequence (5’-3’)**	**Reverse sequence (5’-3’)**
*miR-6862*	CGGGCATGCTGGGAGAGAC	GAACATGTCTGCGTATCTC
*U6 RNA*	CTCGCTTCGGCAGCACAT	TTTGCGTGTCATCCTTGCG
*SphK1*	GCTGGCAGCTTCCTTGAACCAT	GTGTGCAGAGACAGCAGGTTCA
*GAPDH*	GTCTCCTCTGACTTCAACAGCG	ACCACCCTGTTGCTGTAGCCAA

**Primers for qPCR assay.**

**miR-6862 precursor sequence:**

CGAAGCGGGCAUGCUGGGAGAGACUUUGUGAUUUGUCUCCAAAGCCUCACCCAGCUCUCUGGCCCUCUAG

**Mature miR-6862 sequence:**

CGGGCAUGCUGGGAGAGACUUU.

To further verify a direct binding between miR-6862 and *SphK1* mRNA, we created two mutant miR-6862 mimics containing mutations at the binding sites to *SphK1* 3’-UTR (Figure 1H). The two were named as “Mut1-” and “Mut2-” (Figure 1H). The wild-type (WT-) and the two mutant miR-6862 mimics were individually transfected in SH-SY5Y cells (500 nM for 48h). As shown, transfection of the WT-miR-6862 resulted in robust decreases in SphK1 3’-UTR luciferase reporter activity (Figure 1I) and *SphK1* mRNA expression (Figure 1J). Contrarily, the two mutants, “Mut1-” and “Mut2-”, were completely ineffective (Figure 1I, 1J). These results implied that miR-6862 directly binds to and silences SphK1 in SH-SY5Y cells.

We further hypothesized that miR-6862 inhibition could increase SphK1 expression. Therefore, a lentiviral construct encoding premiR-6862 anti-sense (lv-antagomiR-6862) was transduced to SH-SY5Y cells. Stable cells were established with puromycin selection. In stable cells with lv-antagomiR-6862, the mature miR-6862 expression was depleted (over 95% reduction of control cells, Figure 1K). As a result, SphK1 3’-UTR luciferase reporter activity (Figure 1L) and *SphK1* mRNA expression (Figure 1M) were boosted. SphK1 protein elevation was detected as well (Figure 1N) and SphK2 expression was unchanged (Figure 1N). The lentiviral construct encoding anti-sense control sequence, lv-antagomiRC, did not alter miR-6862 and SphK1 expression (Figure 1K–1N). These results showed that miR-6862 directly binds to and silences SphK1 in SH-SY5Y neuronal cells.

### miR-6862 inhibition protects neuronal cells from MPP^+^

**Figure 2:**
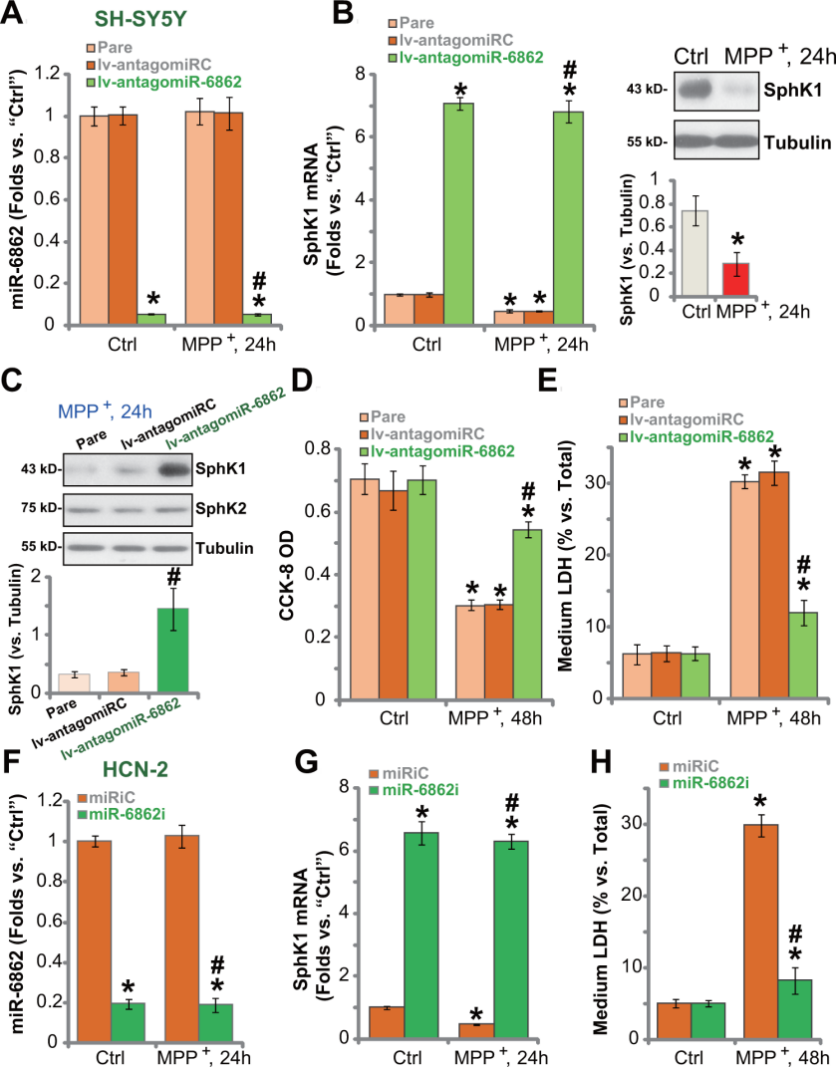
**miR-6862 inhibition protects neuronal cells from MPP^+^.** Parental control SH-SY5Y cells (“Pare”) as well as stable SH-SY5Y cells, expressing the lentiviral construct encoding the anti-sense of premiR-6862 (lv-antagomiR-6862) or the anti-sense control sequence (lv-antagomiRC), were treated with or without MPP^+^ (3 mM); Cells were then cultured for applied time periods, expression of miR-6862 (**A**) and *SphK1* mRNA (**B**) was tested by qPCR assays, with SphK1 protein expression tested by Western blotting analyses (**B**, **C**); Cell viability and death were tested by the CCK-8 assay (**D**) and the LDH release assay (**E**), respectively. HCN-2 neuronal cells were transfected with 500 nM of miR-6862 inhibitor (miR-6862i) or the miR inhibitor control (miRiC) for 48h. Cells were then treated with or without MPP^+^ (3 mM) and cultured for indicated time periods, expression of miR-6862 (**F**) and *SphK1* mRNA (**G**) was tested by qPCR, with cell death examined by medium LDH release assay (**H**). Bars stand for mean ± standard deviation (SD, n=5). * *P* < 0.05 vs. “Ctrl” treatment in “Pare” cells or “miRiC” cells. ^#^ *P* < 0.05 vs. MPP^+^ treatment in “Pare” cells or “miRiC” cells. Experiments in this figure were repeated five times, with the similar results obtained.

Studies have demonstrated that MPP^+^ inhibited SphK1 expression and activity in neuronal cells, mediating cell death [20, 21]. Conversely, forced activation of SphK1 inhibited MPP^+^-induced cytotoxicity [22]. Based on the results in Figure 1, we proposed that SphK1 elevation by miR-6862 inhibition should offer neuronal cell protection against MPP^+^. The qPCR assay results showed that in SH-SY5Y cells, MPP^+^ (3 mM [10]) did not alter miR-6862 expression (Figure 2A). It however downregulated *SphK1* mRNA expression (Figure 2B). SphK1 protein expression was decreased as well (Figure 2B, the right panel). In the lv-antagomiR-6862-expressing SH-SY5Y cells (see Figure 1), mature miR-6862 expression was dramatically downregulated with or without MPP^+^ treatment (Figure 2A). Importantly, *SphK1* mRNA expression increased over seven folds of control even after MPP^+^ stimulation (Figure 2B). Figure 2C confirmed the restoring of SphK1 protein expression in lv-antagomiR-6862-expressing SH-SY5Y cells with MPP^+^ treatment. SphK2 protein expression was again unchanged (Figure 2C).

In line with our previous study [10], MPP^+^ treatment (3 mM, 48h) resulted in robust viability (CCK-8 OD) reduction (Figure 2D) and cell death (increased medium LDH release, Figure 2E) in parental SH-SY5Y cells. MPP^+^-induced cytotoxicity was however largely mitigated in lv-antagomiR-6862SH-SY5Y cells (Figure 2D, 2E). The lv-antagomiRC did not affect MPP^+^-induced cytotoxicity (Figure 2D, 2E).

In HCN-2 neuronal cells, transfection of miR-6862 inhibitor (miR-6862i) downregulated miR-6862 (Figure 2F) and induced *SphK1* mRNA upregulation (Figure 2G) regardless of MPP^+^ treatment (Figure 2F, 2G). MPP^+^-induced HCN-2 cell death, evidenced by increased medium LDH release (Figure 2H), was largely inhibited by miR-6862i. Thus miR-6862 inhibition increased SphK1 expression and protected neuronal cells from MPP^+^-induced cytotoxicity.

### miR-6862 inhibition attenuates MPP^+^-induced apoptosis in neuronal cells

**Figure 3:**
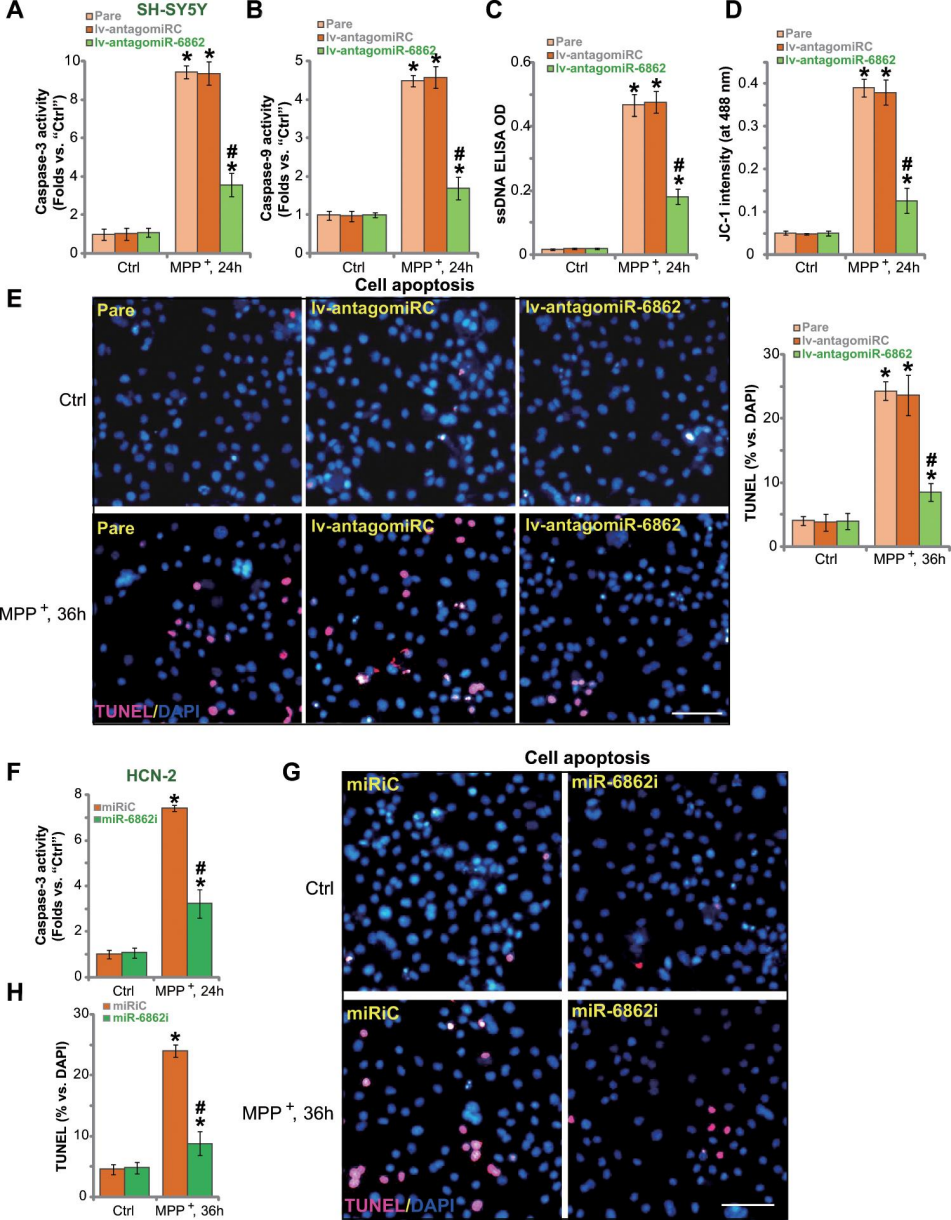
**miR-6862 inhibition attenuates MPP^+^-induced apoptosis in neuronal cells.** Parental control SH-SY5Y cells (“Pare”) as well as stable SH-SY5Y cells, expressing the lentiviral construct encoding the anti-sense of premiR-6862 (lv-antagomiR-6862) or the anti-sense control sequence (lv-antagomiRC), were treated with or without MPP^+^ (3 mM); Cells were then cultured for applied time periods, the caspase-3/-9 activity (**A**, **B**), single strand DNA (ssDNA) contents (**C**) and mitochondrial depolarization (recording JC-1 intensity at 488 nm, **D**) were tested. Cell apoptosis was tested by nuclear TUNEL staining assay (**E**). HCN-2 neuronal cells were transfected with 500 nM of miR-6862 inhibitor (miR-6862i) or the miR inhibitor control (miRiC) for 48h. Cells were then treated with or without MPP^+^ (3 mM) and cultured for indicated time periods, caspase-3 activity (**F**) and cell apoptosis (**G**, **H**) were tested similarly. Bars stand for mean ± standard deviation (SD, n=5). * *P* < 0.05 vs. “Ctrl” treatment in “Pare” cells or “miRiC” cells. ^#^ *P* < 0.05 vs. MPP^+^ treatment in “Pare” cells or “miRiC” cells. Experiments in this figure were repeated five times, with the similar results obtained. Scale bar= 100 μm (**E**, **G**).

Studies have shown that MPP^+^ inhibited SphK1 activity to provoke apoptosis activation in neuronal cells [20, 21]. We therefore tested whether miR-6862 inhibition could affect MPP^+^-induced neuronal cell apoptosis. In parental control SH-SY5Y cells, treatment with MPP^+^ significantly increased the activities of caspase-3 (Figure 3A) and caspase-9 (Figure 3B). It also caused single strand DNA (ssDNA) accumulation that indicates DNA breaks (Figure 3C). Mitochondrial depolarization, or JC-1 green monomers accumulation (increased JC-1 intensity at 488 nm), was detected in MPP^+^-treated SH-SY5Y cells (Figure 3D). Importantly, miR-6862 silencing by lv-antagomiR-6862 inhibited MPP^+^-induced caspase-3/-9 activation (Figure 3A, 3B), ssDNA accumulation (Figure 3C), and mitochondrial depolarization (Figure 3D) in SH-SY5Y cells. TUNEL staining assay results in Figure 3E showed that MPP^+^ induced significant apoptosis activation in SH-SY5Y cells. As TUNEL-positive nuclei ratio was significantly elevated in MPP^+^-treated cells (Figure 3E). Importantly, apoptosis activation by MPP^+^ was largely inhibited by lv-antagomiR-6862 (Figure 3E).

In HCN-2 neuronal cells, transfection of the miR-6862 inhibitor (miR-6862i) largely inhibited MPP^+^-induced caspase-3 activation (Figure 3F) and apoptosis induction (TUNEL staining assay, Figure 3G, 3H). Collectively, miR-6862 inhibition alleviated MPP^+^-induced apoptosis in neuronal cells.

### miR-6862 overexpression augments MPP^+^-induced neuronal cell death

**Figure 4:**
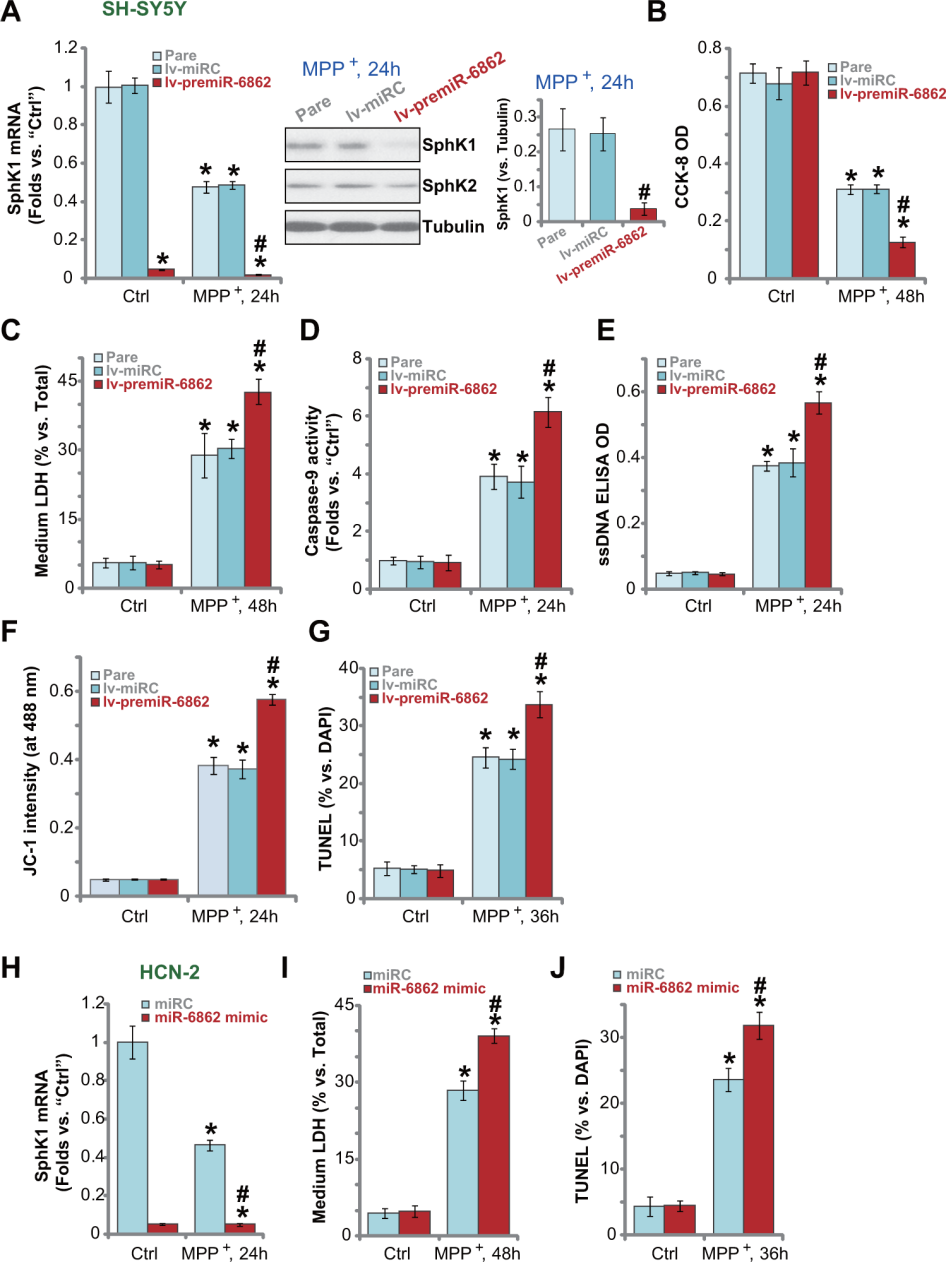
**miR-6862 overexpression augments MPP^+^-induced neuronal cell death.** Parental control SH-SY5Y cells (“Pare”) as well as stable SH-SY5Y cells, with the lentiviral construct encoding the premiR-6862 sequence (lv-premiR-6862) or the nonsense miRNA sequence (lv-miRC), were treated with or without MPP^+^ (3 mM), cells were then cultured for applied time periods, *SphK1* mRNA and protein expression was tested (**A**); Cell viability and death were tested by CCK-8 assay (**D**) and LDH release assay (**E**), respectively; Caspase-9 activation (**D**), single strand DNA (ssDNA) contents (**E**) and mitochondrial depolarization (JC-1 dye assay, **F**) were tested, with cell apoptosis tested by nuclear TUNEL staining assay (**G**). HCN-2 neuronal cells were transfected with 500 nM of miR-6862 mimic or the miR control mimic (“miRC”) for 48h. Cells were then treated with or without MPP^+^ (3 mM) and cultured for indicated time periods, *SphK1* mRNA expression (**H**), medium LDH contents (**I**), and cell apoptosis (by recording TUNEL-positive nuclei, (**J**) were tested. Bars stand for mean ± standard deviation (SD, n=5). * *P* < 0.05 vs. “Ctrl” treatment in “Pare” cells or “miRC” cells. ^#^ *P* < 0.05 vs. MPP^+^ treatment in “Pare” cells or “miRC” cells. Experiments in this figure were repeated five times, with the similar results obtained.

Next, we studied whether miR-6862 overexpression would exert opposite functions and increase MPP^+^-induced neuronal cell death. In SH-SY5Y cells, forced miR-6862 overexpression by lv-premiR-6862 (see Figure 1) aggravated MPP^+^-induced *SphK1* mRNA and protein downregulation (Figure 4A). MPP^+^-induced viability (CCK-8 OD) reduction (Figure 4B) and cell death (by recording medium LDH release, Figure 4C) were intensified in lv-premiR-6862 SH-SY5Y cells. Furthermore, with miR-6862 overexpression, MPP^+^-induced caspase-9 activation (Figure 4D), DNA breaks (ssDNA accumulation, Figure 4E) and mitochondrial depolarization (JC-1 green monomer intensity increase, Figure 4F) were augmented. Forced miR-6862 overexpression enhanced MPP^+^-induced apoptosis activation in SH-SY5Y cells (Figure 4G), which was reflected by increased TUNEL-positive nuclei ratio (Figure 4G).

Transfection of miR-6862 mimic (500 nM) caused substantial *SphK1* mRNA downregulation in HCN-2 neuronal cells with MPP^+^ treatment (Figure 4H). Consequently, MPP^+^-induced cell death (medium LDH release, Figure 4I) and apoptosis (reflected by TUNEL-positive nuclei ratio, Figure 4J) were augmented. Together, miR-6862 overexpression augmented MPP^+^-induced neuronal cell apoptosis.

### SphK1 knockout intensifies MPP^+^-induced neuronal cell death

**Figure 5:**
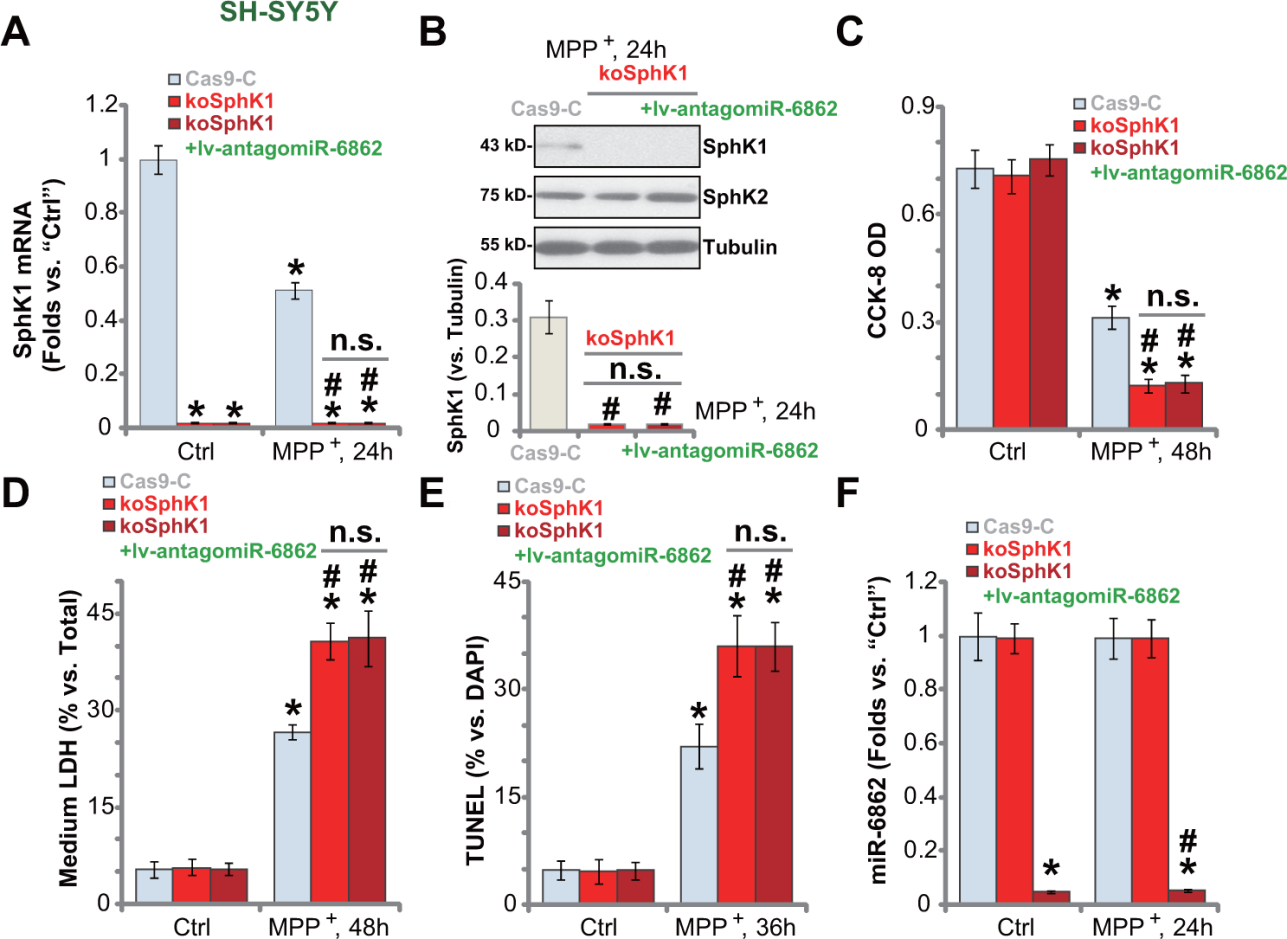
**SphK1 knockout intensifies MPP^+^-induced neuronal cell death.** Stable SH-SY5Y cells expressing the lentiCRISPR-GFP-SphK1-KO construct (“koSphK1” cells) were further transduced with or without the lentiviral construct encoding the anti-sense of premiR-6862 (lv-antagomiR-686), control cells were transduced with lentiCRISPR-GFP empty vector (“Cas9-C”); Cells were treated with or without MPP^+^ (3 mM) and then cultured for applied time periods, *SphK1* mRNA and protein expression was tested (**A**, **B**); Cell viability and death were tested by CCK-8 (**C**) and LDH release (**D**) assays, respectively; Cell apoptosis was examined by nuclear TUNEL staining assay (**E**). miR-6862 expression was shown (**F**). Bars stand for mean ± standard deviation (SD, n=5). * *P* < 0.05 vs. “Ctrl” treatment in “Cas9-C” cells. ^#^ *P* < 0.05 vs. MPP^+^ treatment in “Cas9-C” cells. “n.s.” stands for non-statistical difference. Experiments in this figure were repeated five times, with the similar results obtained.

We further hypothesized that SphK1 knockout should mimic miR-6862 overexpression-induced actions. Therefore, a lentiCRISPR-GFP-SphK1-KO construct (from Dr. Yao at Nanjing Medical University [25]) was transduced to SH-SY5Y cells. Stable cells were established (koSphK1 cells, see Methods). *SphK1* mRNA (Figure 5A) and protein (Figure 5B) expression were depleted in koSphK1 cells. As compared to control cells with empty vector (“Cas9-C”), MPP^+^-induced viability reduction was intensified in koSphK1 SH-SY5Y cells (Figure 5C). Furthermore, in koSphK1 cells, MPP^+^-induced cell death (medium LDH release, Figure 5D) and apoptosis (by recording TUNEL-positive nuclei ratio, Figure 5E) were aggravated. Therefore, CRISPR/Cas9-induced SphK1 KO intensified MPP^+^-induced neuronal cell death.

Importantly, though lv-antagomiR-6862 silenced miR-6862 (Figure 5F) in koSphK1 cells, it failed to offer neuroprotective activity against MPP^+^. Specifically, MPP^+^-induced viability reduction (Figure 5C), cell death (Figure 5D) and apoptosis (Figure 5E) were not alleviated by lv-antagomiR-6862 in koSphK1 SH-SY5Y cells. These results suggested that miR-6862 inhibition protects neuronal cells from MPP^+^-induced apoptosis through upregulating SphK1.

## DISCUSSION

The loss of DA neurons in the midbrain is a key pathological characteristics of PD progression, and it could be due to mitochondrial dysfunction, free radicals (ROS) accumulation, sustained oxidative injury, as well as abnormal protein aggregation and inflammatory lesions [5, 29]. Of particular relevance, mitochondrial dysfunction, causing ROS accumulation and oxidative injury, is one primary cause of DA neuronal cell death [5, 29].

The postmortem brain analyses and animal model studies have revealed that miRNA dysregulation is associated with PD [30, 31] and other neurodegenerative disorders such as Alzheimer's disease and Huntington's disease [32, 33]. Understanding miRNAs and their targeted genes in neurodegenerative diseases should help to identify novel genes for neuronal functions and to reveal the underlying molecular mechanisms underlying these diseases [32, 33]. Since miRNAs regulating genes and pathways that are associated with PD are altered in PD, it is useful in the diagnose of PD and even different PD subtypes [30, 31]. Furthermore, miRNAs are important in regulating neuronal cell death by MPP^+^.

Datta et al., have shown that microRNA-7 (miR-7) prevented MPP^+^-induced cell death in SH-SY5Y cells and differentiated human neural progenitor ReNcell VM cells [34]. In addition, miR-7 downregulated RelA, a member of the nuclear factor κB (NF-κB) family of transcription factors, and augmented neuronal glucose transporter 3 (Glut3) to promote glycolysis in SH-SY5Y cells and ReNcell VM cells [34]. Mao et al., found that miR-21 downregulation was able to attenuate MPP^+^-induced MES23.5 cell death by apoptosis inhibition and reactive oxygen species (ROS) scavenging [35]. In SH-SY5Y cells and primary dopaminergic neurons, astrocyte-derived exosomal miR-200a-3p downregulated MKK4 (mitogen-activated protein kinase kinase 4) and prevented MPP^+^-induced apoptotic cell death [36]. miR-6862 is a relatively novel miRNA with its functions largely unknown. Fuhrmannet al., demonstrated that miR-6862 silenced nuclear receptor coactivator 4 (NCOA4) to inhibit mitochondrial ferritin (FTMT) formation [37]. Zhu et al., reported that miR-6862 expression is dysregulated in lung adenocarcinoma. Here we identified miR-6862 as a novel SphK1-targeting miRNA in neuronal cells. MiR-6862 locates in the cytoplasm of SH-SY5Y neuronal cells and directly binds to *SphK1* mRNA. Ectopic overexpression of miR-6862 decreased *SphK1* 3’-UTR luciferase reporter activity and downregulated its expression in neuronal cells. miR-6862 inhibition, by lv-antagomiR-6862 construct or miR-6862 inhibitor, exerted opposite activity and elevated SphK1 expression in neuronal cells.

SphK1 converts sphingosine to S1P, the bioactive lipids with important functions in cell survival, differentiation, migration, and trafficking [14, 38]. SphK1 downregulation was detected in MPP^+^-treated SH-SY5Y neuronal cells [21]. Exogenous S1P addition would activate SphK1 and inhibited MPP^+^-induced neuronal cell apoptosis [20, 21]. K6PC-5, a SphK1 activator, protected SH-SY5Y neuronal cells from oxygen glucose deprivation/re-oxygenation-induced oxidative stress [22]. Here in SH-SY5Y and HCN-2 neuronal cells, MPP^+^-induced cell death was attenuated by miR-6862 inhibition with either lv-antagomiR-6862 or miR-6862 inhibitor. Conversely, ectopic overexpression of miR-6862 further downregulated SphK1 and augmented MPP^+^-induced neuronal cell apoptosis. Thus, SphK1 elevation by miR-6862 inhibition protected neuronal cells from MPP^+^-induced apoptosis.

Importantly, CRISPR/Cas9-induced SphK1 KO augmented MPP^+^-induced neuronal cell death, which mimicked miR-6862 overexpression-induced activity. Significantly, in koSphK1 SH-SY5Y neuronal cells, exogenously miR-6862 inhibition by lv-antagomiR-6862 failed to alleviate MPP^+^-induced apoptosis. These results indicated that SphK1 should be the primary target of miR-6862 in neuronal cells. Moreover, miR-6862 inhibition-induced neuronal cell protection against MPP^+^ should be through elevating SphK1 expression.

However, the findings of antagomiR-6862-induced neuronal cell protection *in vitro* cannot be simply translated *in vivo*. The *in vivo* experiments are regarded as the gold standard for PD studies [39]. The potential effect of antagomiR-6862 remains to be evaluated in MPTP-induced PD animal model and possibly in genetic-based PD animal models. Also, only established neuronal cell lines, SH-SY5Y and HCN-2, were tested in this study. Our findings have to be further verified in primary DA neurons. In addition, miR-6862 expression was unchanged in MPP^+^-treated cells. Further exploring the expression of miR-6862 in PD postmortem brain tissues and PD animal models would be necessary.

In summary, we identified miR-6862 as a novel SphK1-targeting miRNA. miR-6862 inhibition upregulated SphK1 and protected neuronal cells from MPP^+^-induced damage. Therefore, SphK1 elevation induced bymiR-6862 inhibition could be a novel genetic strategy to protect DA neurons against oxidative injury.

## MATERIALS AND METHODS

### Reagents and antibodies

MPP^+^, puromycin, polybrene and CCK-8 assay kit were obtained from Sigma-Aldrich (St. Louis, Mo). The antibodies were all provided by Cell Signaling Tech (Danvers, MA) and Abcam (Shanghai, China). Lipofectamine 2000 and other transfection reagents were provided by Thermo-Fisher Invitrogen (Shanghai, China). All the primers, sequences, and viral constructs were provided by Shanghai Genechem Co. (Shanghai, China) unless otherwise mentioned.

### Cell culture

Culturing the differentiated SH-SY5Y neuronal cells was reported before [10, 40]. The HCN-2 neuronal cell line was purchased from the Cell Bank of Shanghai Institute of Biological Science (Shanghai, China). HCN-2 cells were cultured in DMEM with 10% FBS.MPP^+^ was dissolved in PBS to obtain 30 mM MPP^+^ stock solution, and was then filtrated and stored in dark at -20° C. Neuronal cells with applied genetic modifications were treated with MPP^+^ (3 mM). This concentration was chosen based on our previous studies [10, 41].

### miR-6862 overexpression or inhibition

GV369 lentiviral constructs encoding the miR-6862 precursor sequence (premiR-6862, sequence listed in Table 2) or the anti-sense sequence (anti-premiR-6862) were synthesized by Genechem (Shanghai, China). Each was transfected in HEK-293T cells along with the lentivirus package plasmids (Genechem) to generate the premiR-6862 expression lentivirus (“lv-premiR-6862”) and the premiR-6862 anti-sense lentivirus (“lv-antagomiR-6862”). Neuronal cells were cultured into six well-tissue plates (at 2 × 10^5^ cells per well)under the polybrene-containing medium, and lentivirus was then added. Puromycin (5.0 μg/mL) was further added to select stable cells, and mature miR-6862 expression (sequence listed in Table 2) was examined by qPCR.

### Transfection of miR mimic

SH-SY5Y and HCN-2 neuronal cells were cultured into six well-tissue plates (at 2 × 10^5^ cells per well) and transfected with 500 nM of the applied miR mimic or miR inhibitor through a Lipofectamine 2000 protocol [42].

### SphK1 3'-UTR activity assay

Briefly, *SphK1* 3’-UTR containing the putative binding sites of miR-6862(at position 113-120) was provided by Dr. Yao [25]. It was inserted into a firefly luciferase reporter vector pGL4.13 (luc2/SV40) [25]. SH-SY5Y neuronal cells were cultured into six well-tissue plates (at 2 × 10^5^ cells per well) and transfected with the plasmid as well as the Renilla luciferase reporter vector and pRL-SV40 [25] (by Lipofectamine 2000). Cells with the applied genetic modifications were subjected to SphK1 3'-UTR luciferase activity assay using a Promega kit [42].

### RNA-Pull down assay

RNA-Pull down was analyzed by a Pierce Magnetic RNA Pull-Down Kit [43, 44]. Briefly, SH-SY5Y neuronal cells were cultured into six well-tissue plates (at 2 × 10^5^ cells per well) and transfected with biotinylated miR-6862 mimic (100 nmol/L) for 48h [44]. The streptavidin-coated magnetic beads were added to total cell lysates to pull-down biotin-captured miR-6862-bound RNA complex [43]. *SphK1 mRNA* expression was tested by qPCR with level normalized (% of input controls).

### Cell viability

Neuronal cells were plated into 96 well-tissue plates (at 3, 000 cells per well). Following the applied MPP^+^ treatment, a CCK-8 assay kit was utilized to test cell viability with the attached protocol. CCK-8’s optical density (OD) was tested at the test-wavelength of 550 nm.

### Quantitative real-time reverse transcriptase polymerase chain reaction (qPCR)

The detailed protocols of qPCR were described in our previous study [10]. Briefly, TRIzol reagents (Sigma) were utilized to extract total cellular RNA, which was then reversely transcribed to cDNA. A TOYOBO ReverTra Ace qPCR kit (Tokyo, Japan) was applied for qPCR under the ABI Prism 7500H fast Real-Time PCR system (Foster City, CA). The methods for mRNA data quantification and normalization to GAPDH were described before [10]. A TransStartTM SYBR Green qPCR Supermix (TransGen Biotech) was utilized to examine miR-6862 expression, and the results were normalized to U6 RNA. The mRNA primers were listed in Table 2.

### Lactate dehydrogenase (LDH) assay

Neuronal cells were plated in six well-tissue plates. Following the applied MPP^+^ treatment, LDH contents were tested by a simple two-step LDH detection kit (Promega, Shanghai, China). LDH contents in the medium were always normalized to total LDH contents.

### Western blotting

Neuronal cells were plated into six well-tissue plates (at 2 × 10^5^ cells per well). Following the applied treatment, cell lysates were obtained. Detailed protocols of Western blotting were described before [10]. Data quantification was performed through ImageJ software (NIH).

### Caspase activity

Neuronal cells were plated into six well-tissue plates (at 2 × 10^5^ cells per well). Following the MPP^+^ treatment, cell lysates were obtained. The relative activities of caspase-3 and caspase-9 were tested by fluorometric caspase assay kits (Beyotime Biotechnology, Wuxi, China) [45] with 20 μg lysates per sample. The caspase-3 or the caspase-9 p-nitroaniline (pNA) absorbance was detected at the wavelength of 405 nm.

Single strand DNA (ssDNA) ELISA assay. Neuronal cells were seeded into six-well plates. Following the applied MPP^+^ treatment, ssDNA contents were tested using a ApoStrandTM ELISA kit (BIOMOL International, Plymouth Meeting, PA).

### TUNEL staining

Neuronal cells were plated into six well-tissue plates (at 2 × 10^5^ cells per well). Following MPP^+^ treatment, cell apoptosis was measured via a TUNEL [terminal deoxynucleotidyl transferase (TdT)-mediated dUTP nick end labeling] *In Situ* Cell Death Detection Kit (Roche, Shanghai, China). TUNEL-positive nuclei percentage (% *vs.* DAPI), from 500 cells per treatment in five random views (1: 100 magnification), was always calculated.

### Mitochondrial depolarization assay

Neuronal cells were plated into 96 well-tissue plates (at 3, 000 cells per well). Following the applied MPP^+^ treatment, cells were stained with the mito-dye JC-1. JC-1 green fluorescence intensity (at 488 nm) was recorded. The representative JC-1 images were presented as well.

### SphK1 knockout (KO)

A lentiCRISPR-GFP construct encoding the small guide RNA (sgRNA) against human SphK1 was provided by Dr. Yao at Nanjing Medical University [25]. SH-SY5Y neuronal cells were cultured into six well-tissue plates and transfected with SphK1-KO construct. FACS-mediated GFP sorting were utilized to select transfected cells. Cells were then distributed into 96-well plates. Single stable cells with SphK1 KO were further screened by qPCR. Control SH-SY5Y cells were transfected with the empty vector [25].

### Statistics

Data were presented as mean ±standard deviation (SD). Statistical differences were analyzed by one-way analysis of variance (ANOVA) followed by multiple comparisons performed with post hoc Bonferroni test (SPSS, 23.0, SPSS Co. Chicago, CA). A two-tailed unpaired T test was utilized (Excel 2007) when comparing the difference between two treatment groups. Values with ***P*** < 0.05 were considered statistically significant.
